# Modified Incision Design for Submental Flap: An Excellent Design Method for the Reconstruction of a Defect after Head and Neck Tumor Resection

**DOI:** 10.1371/journal.pone.0074110

**Published:** 2013-09-11

**Authors:** Fa-yu Liu, Rui-wu Li, Jawad Safdar, Zhen-ning Li, Nan Guo, Zhong-fei Xu, Shu-fen Ge, Jun-lin Li, Shao-hui Huang, Xue-xin Tan, Chang-fu Sun

**Affiliations:** 1 Department of Oromaxillofacial - Head & Neck Surgery, Department of Oral and Maxillofacial Surgery, School of Stomatology, China Medical University, No. 117, Nanjing Bei Jie, Shenyang, Heping District, China; 2 Department of Head and Neck Surgery, Tumor Hospital of Liaoning Province, Shenyang, China; Sapienza, University of Rome, School of Medicine and Psycology, Italy

## Abstract

**Background:**

The usage of submental flap is a good method for head and neck reconstruction, but it has some risk also, such as anatomical variations and surgical errors. In this article, we present a modified incision design for the submental flap.

**Methods:**

We designed a modified submental flap incision method based on the overlap of the incision outline of the submental flap, platysma myocutaneous flap and infrahyoid myocutaneous flap. If we found that the submental flap was unreliable during the neck dissection at the level III, II and Ib areas, the infrahyoid myocutaneous flap or platysma myocutaneous flap was used to replace it. Between 2004 and 2012, we performed 30 cases using this method. As control, 33 radial forearm free flaps were counted. Significant differences were evaluated using the χ^2^ test and Mann-Whitney U. Survival and recurrence were analyzed using the Kaplan-Meier method.

**Results:**

Of the 30 patients, 27 finally received a submental flap, 1 patient received an infrahyoid myocutaneous flap, and 2 patients received a platysma myocutaneous flap. In patients who received the submental flap, the average operation time was 5.9 hours, 2.4 hours shorter than the radial forearm free flap group; the average age was 61.8, 6.1 years older than the radial forearm free flap group; the survival time and recurrence time did not significantly differ with those of the forearm free flap group; and the success rate was higher than traditional methods.

**Conclusions:**

The wider indications, less required time, the similar low risk of recurrence and death as radial forearm free flap, higher success rate than traditional submental flap harvest methods, and ability to safely harvest a submental flap make the modified incision design a reliable method.

## Introduction

The variable surgical defects that can result from head and neck tumor operations necessitate a broad range of surgical reconstructions, ranging from primary closures and pedicle flaps to free tissue transfers. With the advancement of microsurgical techniques over the recent years, free flap techniques are now being used more frequently for head and neck reconstruction, including radial forearm flaps, anterolateral thigh free flaps, rectus abdominis myocutaneous flaps, and latissimus dorsi flaps. However, not all patients are suitable candidates for free flaps. The surgeon should select the proper reconstruction method according to the patients’ need. The points that should be considered prior to selection include patient general physical and mental health, any known co-morbids, the texture and color match of the flap with the recipient region, whether two operational sites are needed and the surgeon’s clinical experience. Furthermore, to optimize the cosmetic and functional outcomes for any given individual surgical wound, the head and neck surgeon must possess a firm grasp of the fundamental techniques as well as the ability to use a reconstructive modality that meets the unique demands of each defect, as ascertained through a thorough defect analysis [1].

The submental flap is one type of axial flap and was first reported by Martin et al in 1993 [2]. It is a time-honored method in head and neck reconstruction that can provide an excellent skin color, thickness and texture match, with cosmetically acceptable scars. However, there are some risk to give up of this flap due to difficult anatomical variations and surgical errors. In this article, we present our experience of using submental flap with a modified incision design. We believe that submental flap raised by this method will be more effective, and also hope this article, including a retrospective analysis of this flap, will be helpful for expanding the awareness and application of this useful flap system.

## Methods

### The Three Flaps’ Surgical Anatomy

#### Submental flap

The submental artery, a constant branch 1 to 1.5 mm in diameter at its origin, arises after the facial artery exits from the submandibular gland. It runs medially on the mylohyoid muscle along the undersurface of the mandible and runs deep (70 percent) or above (30 percent) the anterior belly of the digastric muscle [3]. The flap is drained by the submental vein, which drains into the common facial vein over the submandibular gland [4].

#### The platysma myocutaneous flap

The platysma and the overlying skin are supplied by direct cutaneous arteries measuring 0.5 mm in diameter. The small arteries are branches of the postauricular and occipital arteries in the upper lateral neck, the facial and submental arteries in the upper medial neck, the superior thyroid artery in the middle of the neck, the subclavian artery in the lower medial neck, and the transverse or superficial cervical arteries in the lateral aspect of the neck. These vessels traverse the undersurface of the platysma muscle to provide blood flow to the overlying skin [5].

#### Infrahyoid myocutaneous flap

This flap comprises the sternohyoid, sternothyroid and upper belly of the omohyoid muscle, and the pedicle includes the upper thyroid artery and vein. The artery usually derives from the external carotid artery but may also originate from the carotid bifurcation or the common carotid artery. In most patients, the vein drains into the facial or internal jugular vein [6].

### Modified Incision design and surgical technique

**Figure 1:**
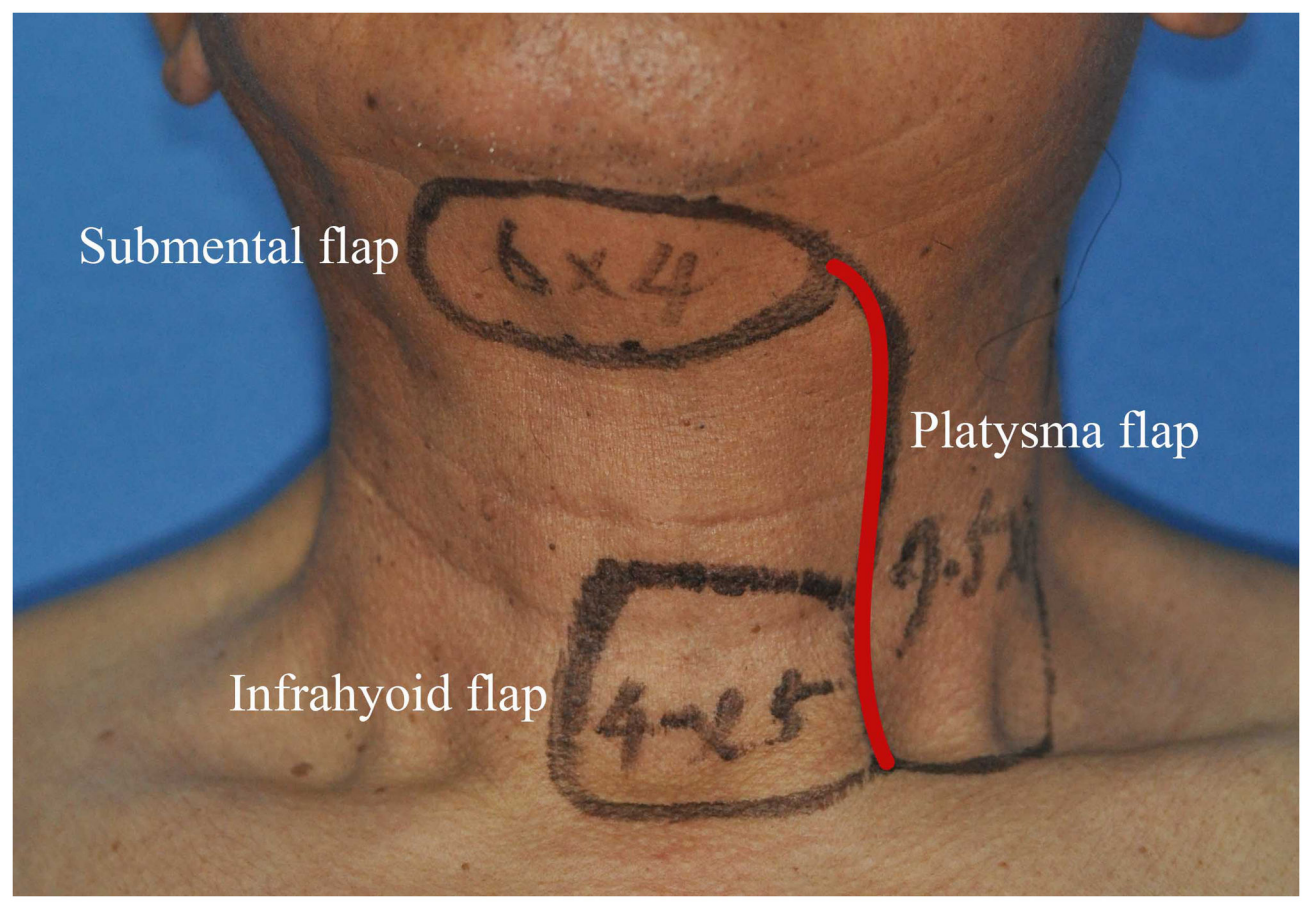
Individual designs of the submental flap, platysma myocutaneous flap and infrahyoid myocutaneous flap. Note the overlap of the incision outline in the middle and upper neck (red line).

**Figure 2:**
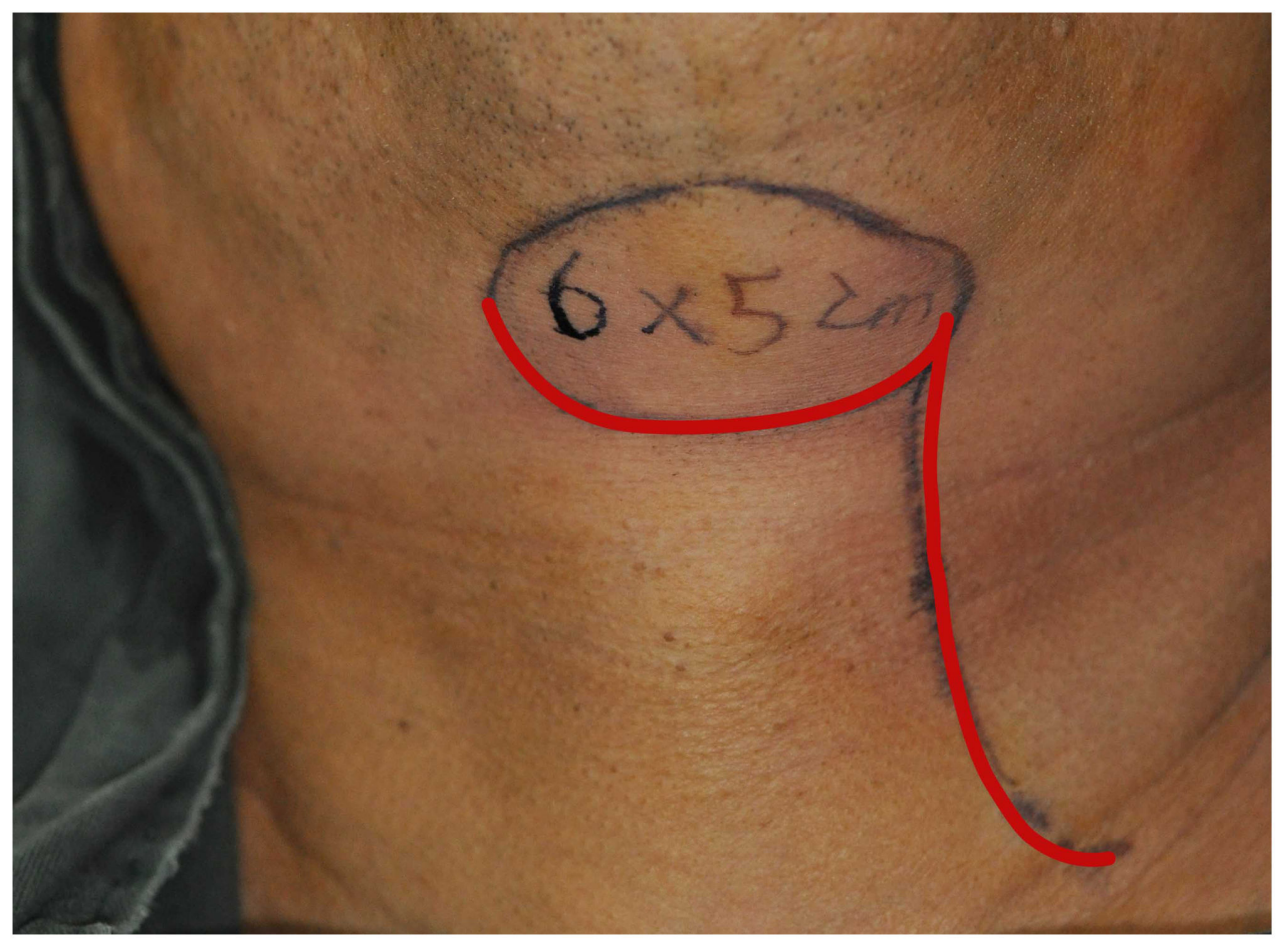
The modified submental flap design.

**Figure 3:**
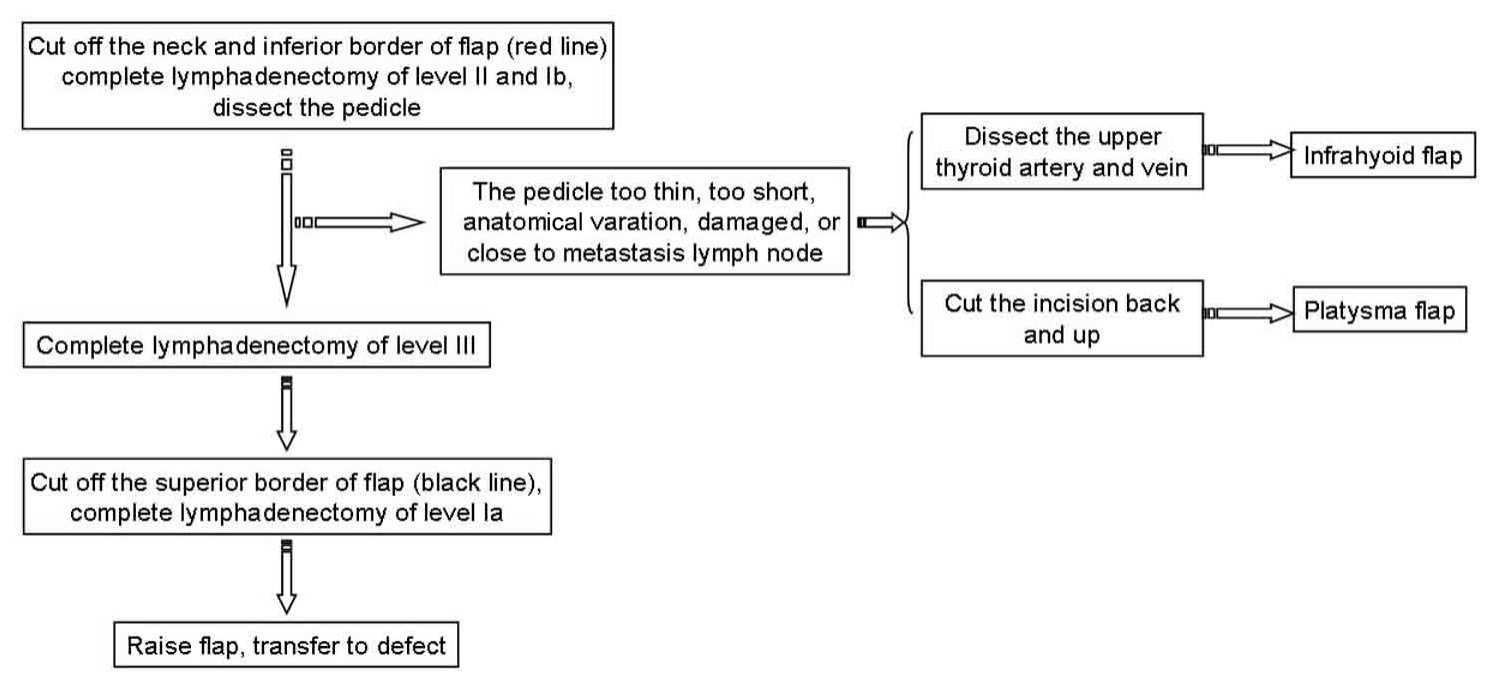
An illustration of the surgical technique.

**Figure 4:**
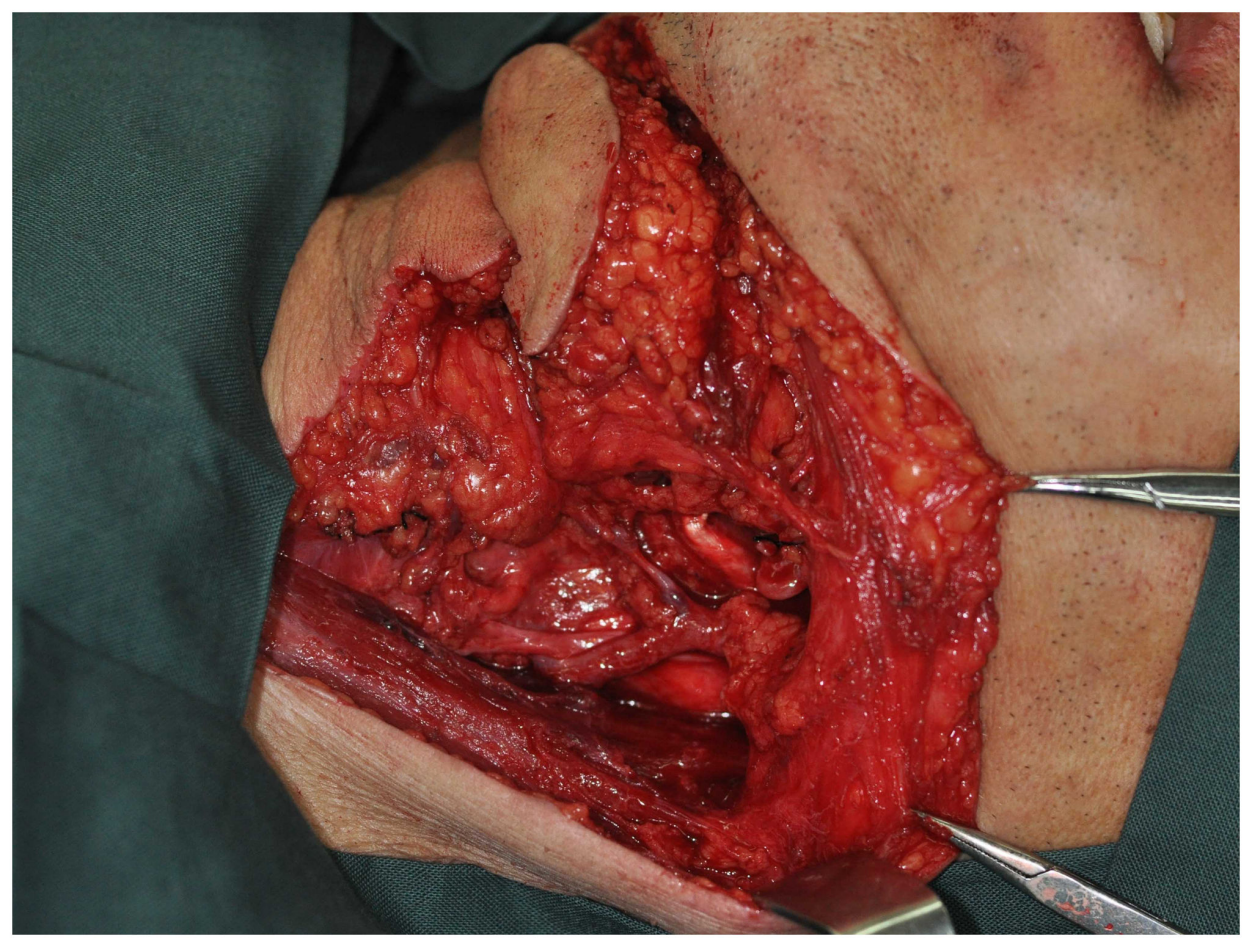
The flap is raised.

**Figure 5:**
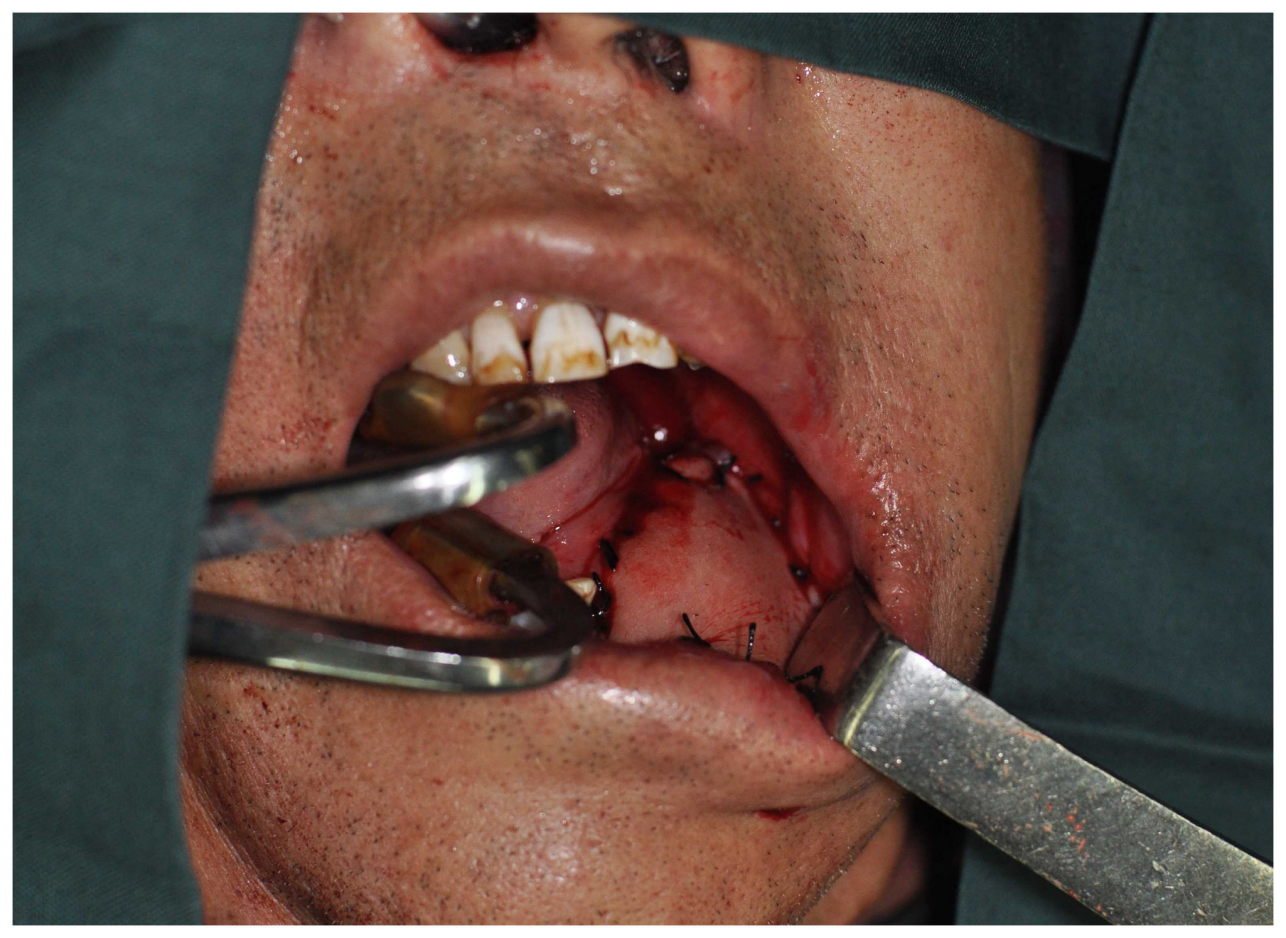
The defect is repaired using a submental flap.

The incision outlines of the submental flap, platysma myocutaneous flap and the infrahyoid myocutaneous flap are shown in Figure 1. Some overlap in the middle and upper neck can be observed (red line). Thus, we design the incision line as shown in Figure 2. Firstly, the neck and inferior border of the flap was incised (red line in Figure 2). Through this incision, the neck skin was undermined in a subplatysmal fashion, and the neck dissection at the level II and Ib areas was completed by traction of the sternomastoid muscle, resecting the submandibular gland, and carefully dissecting the pedicle of the submental flap. If we found that the pedicle was too thin, too short, had anatomical variations, was damaged, or was too close to the metastatic lymph node, the flap was abandoned. The superior thyroid artery and vein were then dissected, and the infrahyoid myocutaneous flap was raised or the incision was made posterior and superior to raise the platysma myocutaneous flap (Figure 3). After the pedicle vessels were identified, the level III lymphadenectomy was easily completed. Then, the superior border of the flap was incised,(black line in Figure 2), the flap was raised, and the level Ia lymphadenectomy was completed (Figure 4). The flap was then transferred to the defect site (Figure 5).

### Patients

#### Ethics statement

All clinical investigations were conducted according to the principles expressed in the Declaration of Helsinki. All the patients provided written informed consent for both the surgical intervention and participation in this research. The individual in this manuscript has given written informed consent (as outlined in PLOS consent form) to publish these case details and photograph. The Ethical Committee of China Medical University has specifically approved this study.

From 2004 to 2012, 30 patients with head and neck tumors were treated using this technique in our hospital. All the patients had not been identified metastasis in level I and II area before the operation. One patient received an infrahyoid myocutaneous flap, and two patients received a platysma myocutaneous flap intraoperatively. The other 27 consecutive patients who received submental flaps were included in this study. The patients’ charts were reviewed for demographic information, tumor location, pathologic diagnosis, comorbid disease, including hypertension, cardiac disease and diabetes mellitus, TNM classification, category of neck dissection, flap size, operation time, complications, functional outcome, time to recurrence, and time to death.

As a control, 33 radial forearm free flaps performed in our hospital during the same period were counted. All these patients have good body state.

### Statistical analysis

The association between complication and comorbid disease in submental flap group was evaluated using the χ^2^ test. The difference in average age and average operation time between submental flap group and radial forearm free flap group were evaluated by Mann–Whitney U test, and the difference in complication rate and comorbid disease rate between the two groups were evaluated using the χ^2^ test. Survival and recurrence were analyzed using the Kaplan-Meier method. All statistical analyses were performed with the software PASW SPSS 18.0. Postoperative function was evaluated according to the method described by Peng et al [7]. A score of 7 points was rated as excellent, 6 and 5 points as better, and fewer than 4 points as poor. A patient with a score of at least 5 points was considered to have an acceptable function.

## Results

As shown in Table 1, the 27 patients who received a submental artery flap ranged in age from 37 to 86 years, with a mean age of 61.8 years. These patients included 18 males and 9 females. The patients exhibited a myriad of comorbidities, including 8 patients with hypertension, 6 patients with cardiac disease, 3 patients with diabetes mellitus, and 3 patients with two conditions. The most frequently encountered surgical defect site was the tongue (n=11), and the most common histologic diagnosis was squamous cell carcinoma (n=22). The size of the flaps ranged from 4 cm × 2.5 cm to 8 cm × 6 cm. Nineteen patients underwent selective node dissection (SND), and 5 patients underwent modified radical neck dissection (MRND). Primary closure was used for all donor sites. Two patients developed full-thickness necrosis of the distal flap tip, and after 10-20 days, both recovered gradually. No patients experienced total flap loss. There was a significant association between this complication (necrosis of the distal flap tip) and cardiac disease (χ^2^=7. 560, p<0.05). The majority of patients (21/27, 77.8%) were satisfied with the results of their reconstructive function, though 3 male patients of them complain some intraoral beard; the remaining patients (6/27, 22.2%) were unsatisfied because their tongue movement was limited to varying degrees. In 15 patients followed up, 9 patients experienced no recurrence within five years (9/15, 60%).

**Table 1:** Twenty-seven consecutive patients who underwent tumor resection and reconstruction with a submental flap.

**Patient**	**Sex**	**Age (y**)	**Tumor location**	**Pathologic diagnosis**	**TNM**	**Flap size**	**Comorbid disease**	**Operation time (h**)	**Time to recurrence (mon**)	**Time to death (mon**)	**Complication**	**Acceptable function**	**Neck dissection**
1	M	69	Tongue, parapharyngeal space	SCC	T4N0M0	6×5	Cd	10	1	15	-	No (LTM)	SND
2	F	68	Tongue, Floor of mouth	SCC	T1N0M0	6×4	-	5.5	No	No	-	Yes	SND
3	M	53	Tongue	SCC	T2N0M0	8×5	-	8	-	-	-	Yes	SND(B)
4	M	51	Tongue	AC	T4N0M0	6×4	-	7.5	6	-	-	No (LTM)	SND
5	F	37	Tongue	SCC	T4N0M0	6×4.5	-	5.66	5	18	-	Yes	MRND
6	F	56	Tongue	SCC	T3N0M0	5×5	Hy	5	-	-	-	No (LTM)	MRND
7	F	40	Tongue	SCC	T2N1M0	5.5×6	-	7.5	No	No	-	Yes	SND
8	F	71	Tongue	SCC	T1N0M0	4×3	Cd	3.83	No	No	Necrosis of distal tip	No (LTM)	SND
9	F	58	Tongue	SCC	T4N0M0	6×4	Dm	10	-	-	-	Yes	SND
10	M	76	Tongue	SCC	T4N0M0	7×5	Cd	7.25	-	-	-	Yes	SND
11	M	86	Sublingual gland	ACC	T3N0M0	7×4	-	2.16	No	53 (natural death)	-	Yes	-
12	M	66	Sublingual gland	ACC	T2N0M0	6×3.5	-	5.25	No	No	-	Yes	SND
13	M	46	Sublingual gland	ACC	T3N0M0	6×4	-	4	No	No	-	Yes	SND
14	M	40	Parotid	ACC	T4N0M0	5×9	Hy	4.5	-	-	-	Yes	MRND
15	M	61	parapharyngeal space	SCC	T1N0M0	4×6	Hy+Cd	7.5	No	No	-	No (LTM)	SND
16	M	55	parapharyngeal space	SCC	T2N0M0	8×6	-	10.08	-	-	-	Yes	SND
17	M	77	Lip	SCC	T3N1M0	6.5×5	Hy+Cd	4.83	-	-	-	Yes	MRND(B)
18	M	74	Lip	SCC	T3N1M0	2.5×4	-	7.66	No	No	-	Yes	SND(B)
19	F	80	Lip	SCC	T2N0M0	3×3	Hy	3.58	-	-	-	Yes	-
20	M	51	Floor of mouth, Tongue	SCC	T4N1M0	7×5	Dm	3.66	3	30	-	No (LTM)	SND
21	M	70	Floor of mouth	SCC	T2N0M0	4×6	Hy+Dm	4.45	-	-	-	Yes	SND
22	M	75	Floor of mouth	SCC	T4N0M0	6.5×4.5	Cd	5	15	55	Necrosis of distal	Yes	MRND
23	M	61	Face	SCC	T4N0M0	6×4	-	5			-	Yes	-
24	M	43	Buccal mucosa	SCC	T3N0M0	6×5	-	4.75	No	No	-	Yes	SND
25	M	63	Buccal mucosa	SCC	T2N0M0	5×11	-	4	-	-	-	Yes	SND
26	F	66	Alveolar ridge	SCC	T4N0M0	4×3	Hy	3.5	-	-	-	Yes	SND
27	F	76	Alveolar ridge	SCC	T2N0M0	4×6	Hy	5	28	30	-	Yes	SND

ACC: adenoid cystic carcinoma. SCC: squamous cell carcinoma. AC: adenocarcinoma. Hy: hypertension. Dm: diabetes mellitus. Cd: cardiac disease. SND: selective neck dissection. MRND: modified radical neck dissection. LTM: limitation of tongue movement. B: bilateral neck

Table 2 shows the comparison between the submental flap group and the 33 radial forearm free flaps. The operation time of patients in the submental flap group who underwent unilateral neck dissection (21 patients) was 5.904 hours, which was 2.428 hours shorter than the radial forearm free flap group (8.332 hours, 22 patients, p<0.05). The average patient age in the submental flap group was about 6 years older than the radial forearm free flap group (no significant difference, p>0.05). The comorbid disease rate was nearly twice that of the radial forearm free flap group (no significant difference, p>0.05), and the complication rate was similar (7.4% and 6.1%).

**Table 2:** Comparison of operation-related factors between the submental flap and radial forearm free flap groups.

	Submental flap	Radial forearm free flap	P
Average age (y)	61.818	55.727	0.071 (Mann-Whitney U=324)
Comorbid disease rate	51.9% (14/27)	27.3% (9/33)	0.065 (χ^2^=3.795)
Average operation time (h, with unilateral neck dissection)	5.904	8.332	0.001* (Mann-Whitney U=88.5)
Complication rate	7.4% (2/27)	6.1% (2/33)	1.000 (χ^2^=0.043)

* P<0.05

**Figure 6:**
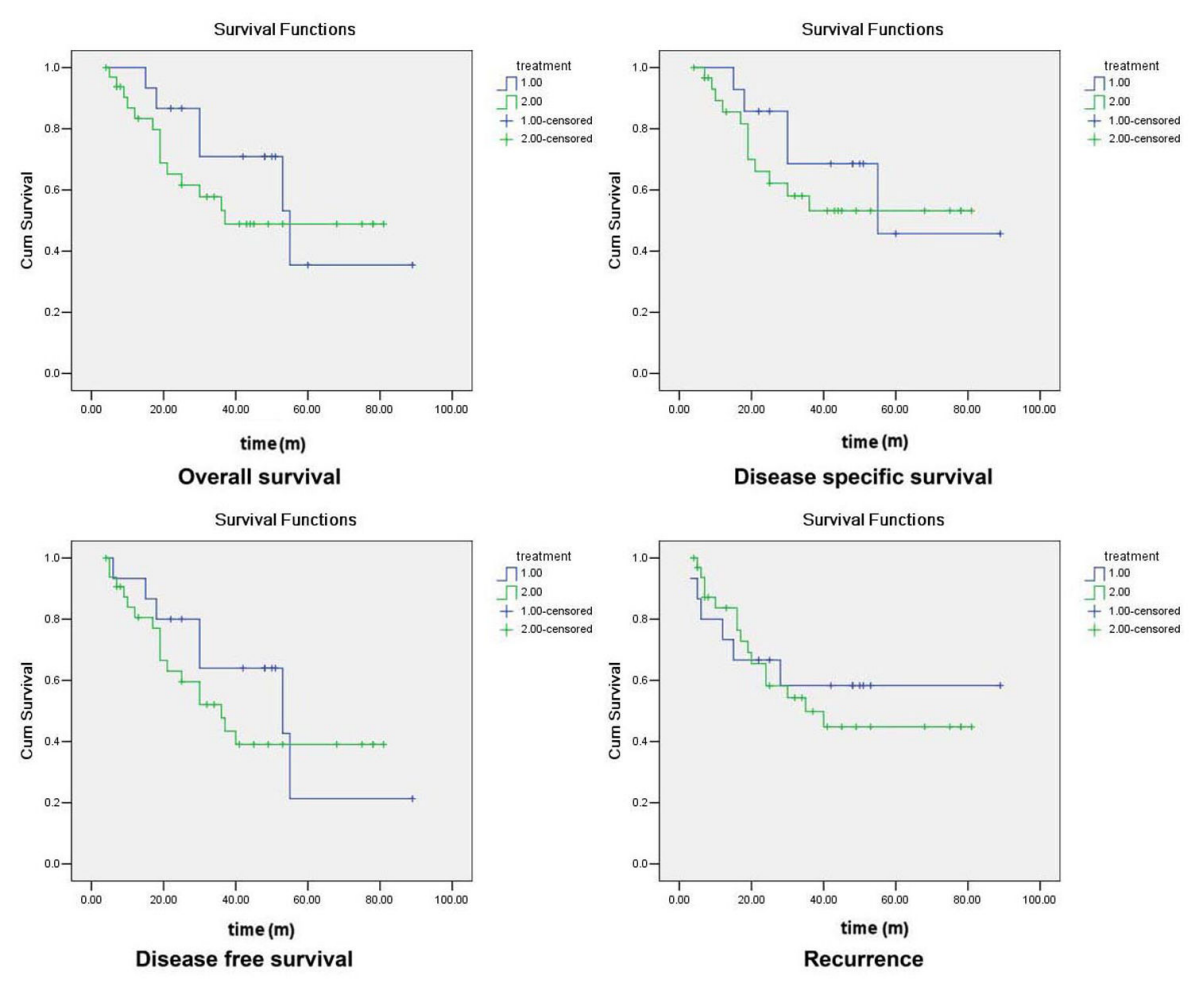
The survival and recurrence graphs of the submental flap and radial forearm free flap group. (1: submental flap group, 2: radial forearm free flap group. Kaplan-Meier method).

Figure 6 shows the survival and recurrence curves of the submental flap patients group and the radial forearm flap patients group. The overall survival time of the submental flap group was 57.627±8.569 months, the disease-specific survival time was 60.757±9.503 months, the disease-free survival time was 49.427±5.529 months, and the recurrence time was 56.983±10.119 months. They all have no significant difference with those of the radial forearm free flap group (Table 3).

**Table 3:** Comparison of survival time and recurrence time between the submental flap and radial forearm free flap groups (Kaplan-Meier test).

	Submental flap	Free flap	P
Overall survival time (m)	57.627±8.569	49.774±6.004	0.439
Disease-specific survival time (m)	60.757±9.503	52.134±6.200	0.478
Disease-free survival time (m)	49.427±5.529	44.563±5.787	0.463
Recurrence time (m)	56.983±10.119	47.158±6.072	0.703

## Discussion

Defects of the head and neck tumors present a significant reconstructive challenge to surgeons, requiring a flap with suitably matched contour, color, and tissue texture [8]. As one type of locoregional flap, the submental flap not only meets the requirements listed above but also requires no donor region, reduces the surgical risk to high-risk patients, such as those with comorbid diseases (e.g., hypertension, diabetes, and old age). In our cases, the average age was 61.8 years, and 14 patients had one or two comorbid diseases (14/27, 51.9%). Among the 33 patients who underwent radial forearm free flap reconstruction in our hospital during the same period, the average age was 55.7 years, and only 9 patients had comorbid diseases (9/33, 27.3%). Although there was no significant difference (p>0.05), these data also imply that the submental flap has wider indications than the radial forearm free flap. In patients in the submental flap group who underwent unilateral neck dissection (21 patients), the average operation time was 5.904 hours, while it was 8.332 hours in radial forearm free flap group patients who underwent unilateral neck dissection (22 patients). The difference between groups was significant (p<0.05), indicating that the submental flap is faster and more easily performed. Of the 27 submental flaps performed, 2 patients experienced full-thickness necrosis of the distal flap tip (2/27, 7.4%), while 2 of the 33 radial forearm free flap patients experienced total flap loss (2/33, 6.1%). Although the complication rates of the two groups were similar, the impact of the submental flap was often less, with no total flap loss. Furthermore, we hypothesize that the low complication rate of the free flaps may be partially due to their strict indications.

To submental flap, there may be a concern about oncological results in some literature [9]. In our research, there have no significant difference between radial forearm free flap group and modified submental flap group in overall survival time, disease-specific survival time, disease-free survival time and the recurrence time, suggesting submental flap will not bring oncological problem if only by right method.

All the comparison demonstrated that the submental flap raised by this method will be a effective complementary of free flap, with some advantage the free flap have no. It should be mentioned that the submental flap may bring intraoral beard to male patients, which should be considered during preoperative design.

Despite these advantages, the submental flap has received lack of attention in recent years, primarily because of the complicated anatomy, difficult for neck dissection and the concern about oncological result.

The pedicle of this flap includes the submental artery and vein, which drain to the facial artery and vein. However, the facial artery and vein do not accompany all the way, and divide at the posterior border of the submandibular gland, with many smaller branches travelling through the gland and fibrofatty tissue. Therefore, it is easy to damage the two vessels when dissecting the pedicle, and this situation is difficult to redeem because the operation has already been half completed.

In our modified design, before the operation, there are two other alternatives to the submental flap. Therefore, if the pedicle is not reliable, the flap can be converted without any other supernumerary incision. In our study of thirty patients, two were found damaged or unreliable submental vessel pedicles intraoperatively and converted to an infrahyoid myocutaneous flap and a platysma myocutaneous flap; both cases obtained good results. This may be the reason that only 2 of the 27 patients who received submental artery flaps experienced full-thickness necrosis of the distal flap tip (2/27, 7.4%), which is lower than the 33.3% (2/6) reported by Lee et al [10] and the 20% (2/10) reported by Patel et al [11]. It should be mentioned that the two cases of distal flap tip necrosis of the submental flap were associated with cardiac disease (p<0.05). It is different with the cervicofacial and cervicothoracic rotation flap complications were previously reported, which were associated with hypertension (p<0.05) [12].

Neck dissection is not more difficult using the modified method, although the incision creates less of a wound than the typical “T”-shape incision, with only a vertical incision in the neck. In our cases, 19 SNDs and 5 MRNDs were easily performed, using traction of the sternocleidomastoid muscle.

**Figure 7:**
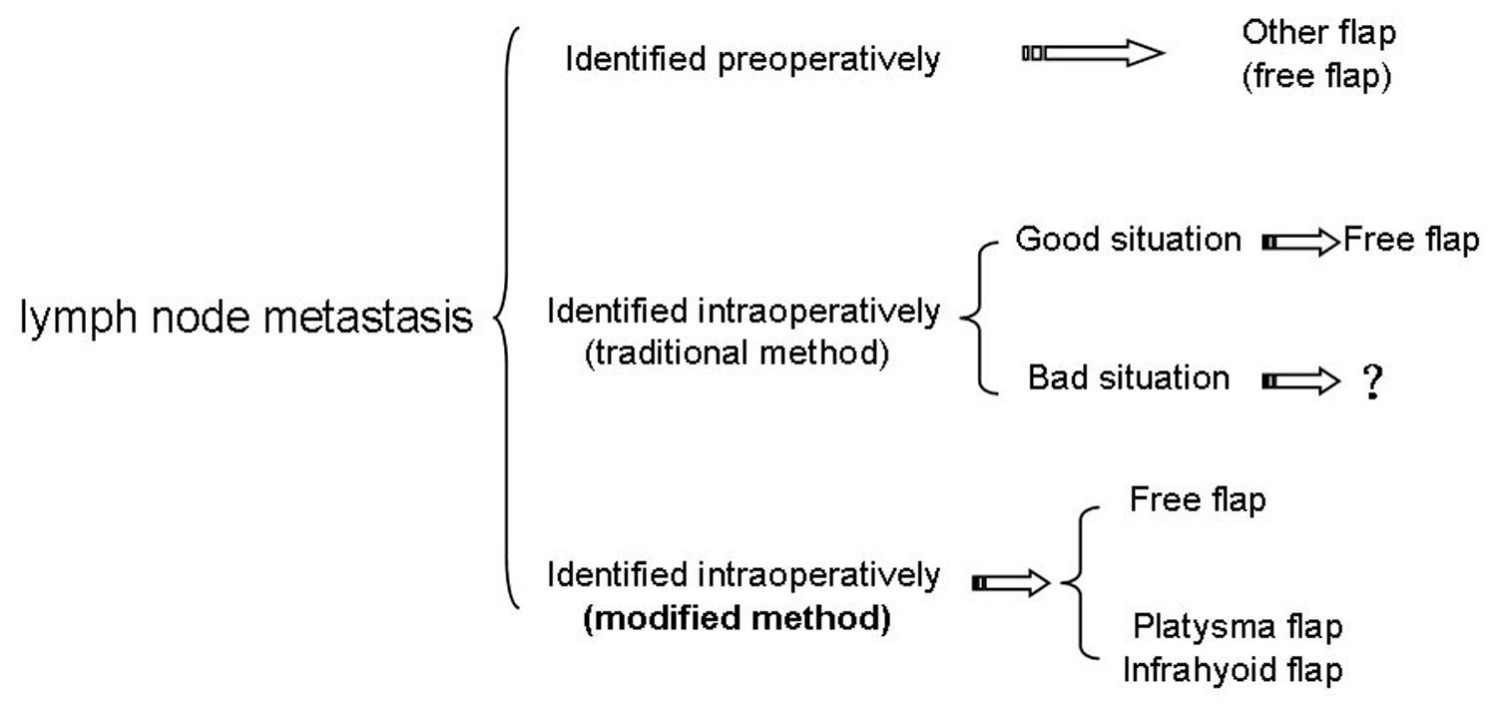
The choice of the reconstruction method in a patient with lymph node metastasis.

The most common tumor in head and neck region is squamous cell carcinoma. In our 27 cases, there are 23 cases of squamous cell carcinoma. Therefore, emerging lymph node metastasis is possible, particularly in the submental region, submandibular region and upper deep cervical region, where the submental flap is located. If metastasis is identified before surgery, a synthetic free flap can be considered. However, when lymph node micrometastasis is observed intraoperatively close to the pedicle, and the submental artery flap is raised, it is unclear how to proceed. Amin et al advised that the surgeon should abandon the submental flap and convert to another reconstructive option [9]. However, using traditional incision methods, the possible neck regional flaps are all destroyed, and the only method possible is the free flap. To patients that associated with a poor condition who cannot endure a free flap, the choice is difficult. Using the modified method, the platysma myocutaneous flap and infrahyoid myocutaneous flap are designed preoperatively, and protected intraoperatively until the submental flap clearly identified. Therefore, when we find the submental flap unreliable, we have no hesitate to abandon it, performing the replacements without further injury (Figure 7). In our cases, one patient was converted to a platysma myocutaneous flap intraoperatively because a metastatic lymph node was adhered to the submental artery, and the result was good: no recurrence within five years. This suggested that this modified method can even be used in suspicious metastasis patient. Of course, it will be better to be used in trauma or benign tumor patients.

**Figure 8:**
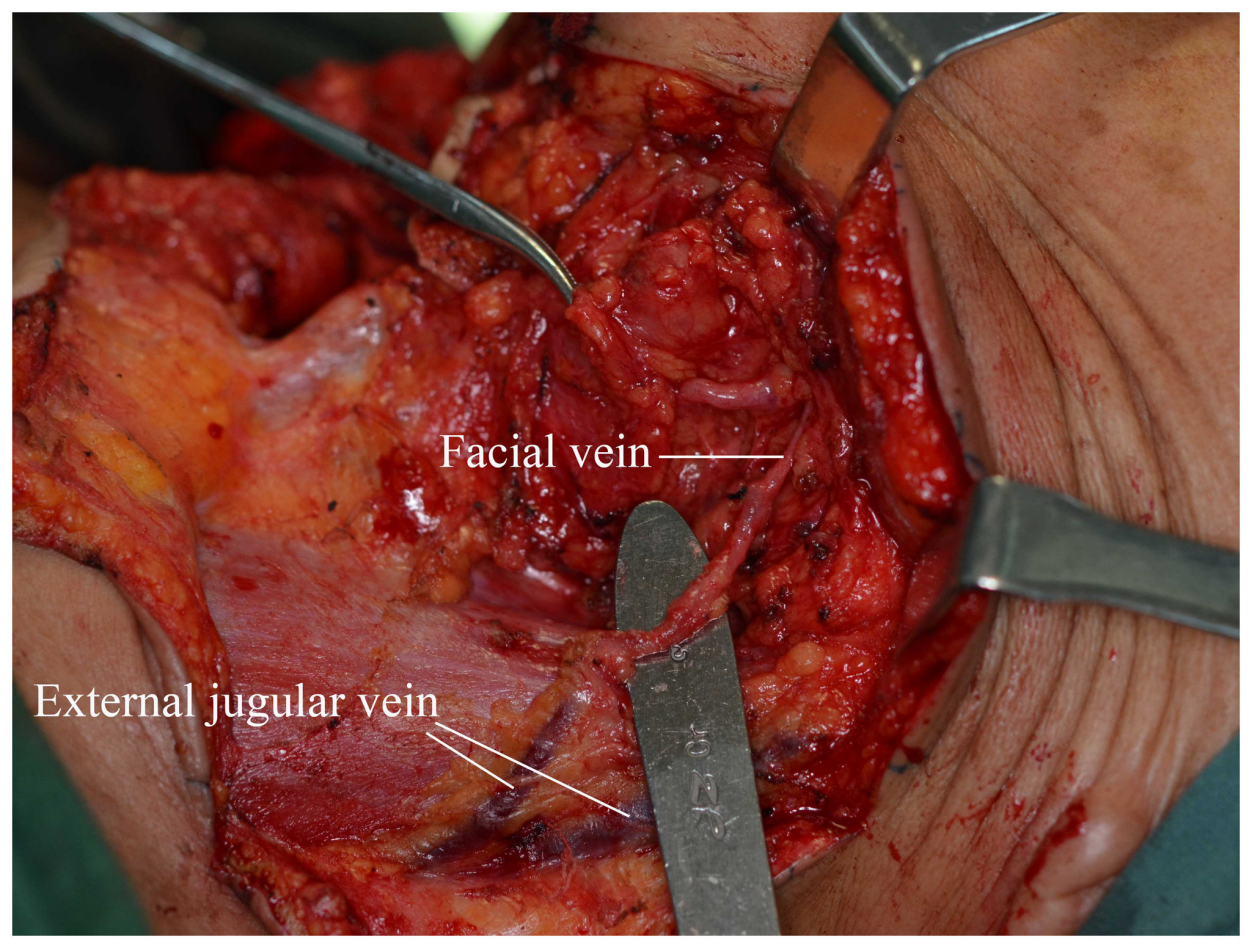
The facial vein drains into the external jugular vein.

Patel et al have suggested raising the submental flap before the lymphadenectomy. They first removed the submandibular gland and dissected the vessel a short distance to the lateral border of the mylohyoid muscle and then raised the flap from the contralateral side, finally dissecting the neck [11]. We believe that this method cannot ensure an oncologically safe procedure because the neck lymph node is not “en bloc” dissected. Furthermore, it is also not optimal to expose the pedicle to the air for an extended time period. Amin et al reported that they used to start the neck dissection, taking extreme caution to preserve the facial vessels, followed by harvesting the flap [9]. However, the blood supply of the submental flap has anatomical variations. The facial vein, which receives blood from the submental vein and usually drains to the internal jugular vein, is found to occasionally drain into the external jugular vein (9%, Figure 8) [13] and cervical anterior vein. And the two veins are often damaged during neck dissection without special protection. Moreover, when we simply performed the lymphadenectomy firstly, it was also very easy to damage the perforator of the upper thyroid artery and vein; if they are damaged, converting to infrahyoid myocutaneous flap is no longer possible. If the transverse incision of the neck is too long, the platysma myocutaneous flap is also no longer possible. Using the modified method, we completed the lymphadenectomy of level II and Ib and dissected the submental artery and vein simultaneously. After the pedicle vessels are clearly identified, the lymphadenectomy can be easily completed without concern of damaging important vessels. Therefore, the operation is short and safe.

There have another modified method about harvesting submental flap, which is “sandwich–the-vessel” method [9,11,14]. In this method, the mylohyoid is included with the flap. At the midline, the dissection is carried deeper into Ia down to the mylohyoid muscle. By including the mylohyoid, the submental artery and the perforating vessels remain protected in this delicate area. We believe, in malignant tumor, especially in squamous cell carcinoma, this “sandwich-the-vessel” method cannot ensure the thorough dissection of the lymph node. Therefore, we insist that the flap should be raised at the subplatysmal plane, up the mylohyoid muscle, leaving the fibrofatty tissues of level Ia for neck dissection, taking care near the vessel offshoot at the medial border on the anterior belly of the digastric muscle. The results showed, necrosis rate of the distal flaps in our cases (7%, 2/27) was no higher than the “sandwich-the-vessel” method (10%, 1/10). This suggests that the modified method can also maintain the success rate of the submental flap, with considering oncological result.

## Conclusions

The modified incision design is a versatile and reliable method to raise a submental flap, which ensures that this flap has wider indications, and requires less time than radial forearm free flap, easier to perform and higher success rate than traditional submental flap harvest methods, and the similar lower risk of recurrence and death as radial forearm free flap. It will make submental flap an effective complementary of free flap.
